# Circ_0083964 knockdown impedes rheumatoid arthritis progression via the miR-204-5p-dependent regulation of YY1

**DOI:** 10.1186/s13018-022-03353-5

**Published:** 2022-12-22

**Authors:** Lei Xiang, Wendi Yang, Feng Wang, Gaozhan Liu

**Affiliations:** Department of Rheumatology, Xiangyang Central Hospital, Affiliated Hospital of Hubei University of Arts and Science, No. 136, Jingzhou Street, Xiangcheng, Xiangyang City, 411000 Hubei Province China

**Keywords:** RA, circ_0083964, miR-204-5p, YY1

## Abstract

**Background:**

Rheumatoid arthritis (RA) is a chronic inflammatory disease. Abnormal proliferation and inflammation of fibroblast-like synoviocytes (FLSs) are the main pathological features of the disease. Accumulating studies have identified that circular RNAs (circRNAs) were involved in the progression of RA. Our study was to assess the function and mechanism of circ_0083964 in RA.

**Methods:**

Quantitative real-time polymerase chain reaction (qRT-PCR) and western blot were utilized to test the level of circ_0083964, miR-204-5p and YY1. Counting Kit-8 (CCK-8) assay, EdU assay, flow cytometry, transwell assay and wound-healing assay were utilized to test cell viability, proliferation, apoptosis, invasion and migration. Cell inflammation was estimated with enzyme-linked immunosorbent assay (ELISA) kits. Dual-luciferase reporter assay and RNA immunoprecipitation (RIP) assay were employed to identify the target relationship between miR-204-5p and circ_0083964 or YY1.

**Results:**

Circ_0083964 was highly expressed in RA synovial tissues and RA-FLSs. Circ_0083964 downregulation constrained proliferation, metastasis and inflammation and facilitated apoptosis in RA-FLSs. Furthermore, circ_0083964 served as a sponge of miR-204-5p, and rescue experiments proved that miR-204-5p deficiency overturned the suppressive impacts of circ_0083964 silencing on RA-FLSs progression. Additionally, we also verified that YY1 could be targeted by miR-204-5p, and its overexpression rescued the repressive impact of miR-204-5p introduction on RA-FLSs development. Besides that, we revealed that circ_0083964 mediated YY1 expression by regulating miR-204-5p.

**Conclusion:**

Circ_0083964 inhibition confined RA development by sponging miR-204-5p to hamper the YY1 level, which will provide a theoretical basis for the treatment of RA.

**Supplementary Information:**

The online version contains supplementary material available at 10.1186/s13018-022-03353-5.

## Highlights

1. Circ_0083964 was overexpressed in RA synovial tissues and RA-FLSs.
2. Circ_0083964 inhibition repressed cell growth, invasion, migration and inflammatory response in RA-FLSs.
3. Circ_0083964 functioned as a molecular sponge of miR-204-5p.
4. YY1 acted as a miR-204-5p target.

**Supplementary Information:**

The online version contains supplementary material available at 10.1186/s13018-022-03353-5.

## Introduction

Rheumatoid arthritis (RA) is an autoimmune disease characterized by chronic inflammation [1]. Although the early diagnosis and treatment of RA has improved in recent years, it is not satisfactory for most patients with a long course of disease [2, 3]. Multiple studies have disclosed that fibroblast-like synoviocytes (FLSs) are involved in the progression of RA by interacting with immune cells and secreting multiple pro-inflammatory cytokines [4, 5]. More importantly, accumulating evidence has shown that inflammatory cytokines can activate RA-FLSs and endowing FLSs with tumorlike properties [6]. Therefore, understanding the mechanism underlying the tumorlike characteristics of RA-FLSs and looking for new therapeutic targets of RA will contribute to exploring potential therapeutic methods for RA.

Circular RNAs (circRNAs) are newly discovered noncoding RNAs (ncRNAs) with circular structures, and circRNAs have pivotal research value in the development of many human diseases because of their high stability [7]. In the past decade, the rapid development of high-throughput sequencing and bioinformatics led to a dramatic increase in the study of circRNAs [8]. At present, multiple circRNAs have been found to participate in the regulation of RA occurrence [9–11]. In addition, studies have also identified that circRNAs can sponge microRNAs (miRNAs) to regulate the level of target genes, ultimately mediating the progression of RA [12]. For example, Zhi et al. [13] showed that circ_AFF2 deficiency retarded the proliferation and inflammation of RA-FLSs by sponging miR-375 to reduce TAB2 expression. Cai et al. [14] reported that interference of circ_0088194 restrained the RA progression via mediating miR-766-3p/MMP2 axis. Circ_0083964, also known as circ-ASH2L, has been shown to be involved in increasing the proliferation and metastatic capacity of RA-FLSs by decreasing HIPK2 expression through absorbing miR-129-5p [15]. However, the underlying mechanisms of circ_0083964 in RA are still poorly understood. In addition, because the disease involves a complex network of genes regulation, it is necessary to clearly elucidate the potential mechanism of circ_0083964 in RA.

Numerous studies have shown that circRNAs influence cell biological process by serving as miRNA sponges [16]. MiRNAs have been reported to be involved in the development of musculoskeletal-related diseases [17, 18]. We predicted that circ_0083964 and miR-204-5p may have a targeted association through StarBase software. The study executed by Xiao et al. [19] found that miR-204-5p was decreased in RA synovial tissues, and the transfection of miR-204-5p curbed proliferation and inflammation, while promoting apoptosis of RA-FLSs. Nevertheless, the mechanism of miR-204-5p in the progression of RA has not been fully elucidated. Additionally, Bioinformatics software predicted the association between YY1 and miR-204-5p. Wang et al. [20] claimed that miR-410-3p could confine the proliferation of RA-FLSs by reducing the level of YY1. Here, we will ulterior determine the role of miR-204-5p/YY1 in RA development.

Hence, our aim was to reveal the role of circ_0083964 in RA. Then, the relationship between circ_0083964, miR-204-5p and YY1 was estimated.

## Materials and methods

### Clinical tissue samples

Synovial tissues were acquired from 21 RA patients who underwent knee replacement surgery at Xiangyang Central Hospital, Affiliated Hospital of Hubei University of Arts and Science. Healthy synovial tissues were harvested from 17 volunteers with traumatic knee disease and no other systemic disease. The study was permitted by the Ethical Committee of Xiangyang Central Hospital, Affiliated Hospital of Hubei University of Arts and Science. Written informed consent has been signed by all participators.

### Cell culture and transfection

The FLSs and RA-FLSs were obtained from the synovial tissues of RA patients and normal volunteers, individually. In brief, sample tissues were cut and digested with collagenase (Invitrogen, Carlsbad, CA, USA) for 4 h. Cells were then obtained by centrifugation, followed by DMEM (Invitrogen) containing 10% FBS (Invitrogen) that was utilized to culture cells at 37 °C with 5% CO_2_. Small interference RNA for circ_0083964 (si-circ_0083964) and its control si-NC, mimic or inhibitor for miR-204-5p (miR-204-5p, anti-miR-204-5p) and related controls (miR-NC and anti-miR-NC), YY1 overexpression plasmid (YY1) and negative control (pcDNA) were bought from RiboBio (Guangzhou, China). Lipofectamine 3000 (Invitrogen) was applied for cell transfection.

### Quantitative real-time polymerase chain reaction (qRT-PCR)

TRIzol reagent (Invitrogen) was applied to collect total RNA from synovial tissues and cells. The synthesis of cDNA from total RNA was executed with a specific RT-PCR kit (Invitrogen). Subsequently, SYBR Green (Invitrogen) was used to conduct a qRT-PCR experiment and the data were calculated by utilizing the 2^−ΔΔCt^ method. Primer sequences were exhibited in Table 1. GAPDH or U6 was applied as an endogenous reference. Total RNA was exposed to 4 U/μg RNase R (GENESEED, Guangzhou, China) at 37 °C for 2 h, and qRT-PCR was performed to investigate the level of circ_0083964 and GAPDH. Random primers or Oligo (dT)18 primers were utilized for reverse transcription assay, then the expression of circ_0083964 and GAPDH was obtained by qRT-PCR.

**Table 1:** Primer sequences used in qRT-PCR

Name		Primers for PCR (5′-3′)
hsa_circ_0083964	Forward	CTGGCTATGGACAGGGAGAC
	Reverse	TAGCCCTTCTCTCCAACCAC
miR-204-5p	Forward	GTATGAGTTCCCTTTGTCATCCT
	Reverse	CTCAACTGGTGTCGTGGAG
miR-211-5p	Forward	GTATGAGTTCCCTTTGTCATCCTT
	Reverse	CTCAACTGGTGTCGTGGAG
miR-142-3p	Forward	GTATGAGTGTAGTGTTTCCTACTTT
	Reverse	CTCAACTGGTGTCGTGGAG
YY1	Forward	GGGCCCTTTGTCCTGGATAC
	Reverse	GTGGATGAGACCTAGCCAGC
GAPDH	Forward	GGAGCGAGATCCCTCCAAAAT
	Reverse	GGCTGTTGTCATACTTCTCATGG
U6	Forward	CGCTTCACGAATTTGCGTGTCAT
	Reverse	GCTTCGGCAGCACATATACTAAAAT

### Cell counting Kit-8 (CCK-8) assay

Cell counting Kit-8 (CCK-8; Solarbio, Beijing, China) was used to estimate cell viability. Shortly, RA-FLSs were cultured into 96-well plates. After 48 h, CCK-8 solution was applied to incubate RA-FLSs for another 3 h, the optical density value was gauged by a microplate reader (Bio-Rad, Hercules, CA, USA).

### 5-ethynyl-2′-deoxyuridine (EdU) assay

The proliferation of RA-FLSs was evaluated by an EdU detection kit (Beyotime, Shanghai, China). Shortly, EdU medium was utilized to incubate RA-FLSs for 3 h, Next, RA-FLSs were then fixed, permeabilized and stained with DAPI (Beyotime). Finally, a fluorescence microscope (Leica, Wetzlar, Germany) was utilized to estimate EdU-positive cells.

### Flow cytometry

RA-FLSs were collected and then washed with PBS. Subsequently, RA-FLSs were stained with Annexin V-FITC and PI (Solarbio) for 20 min. After that, cell apoptosis was quantified via a flow cytometer (BD Bioscience; San Jose, CA, USA).

### Transwell invasion assays

Cell invasion was investigated by a transwell 24-well chamber (Solarbio). The upper chamber was pre-treated with Matrigel (Corning, Cambridge, MA, USA). Serum-free medium and RA-FLSs were resuspended and then seeded into the upper chamber, and the lower chamber was supplemented by a complete medium. After 24 h, cells were stained with 0.1% crystal violet (Solarbio) and then photographed to assess the number of invaded cells.

### Wound-healing assay

Cells were seeded into 12-well plates, and wounds were scratched with a 10 μL sterile pipette tip when the RA-FLSs reach 95–100% fusion. After washing with PBS, cells were incubated for 24 h under the serum-free medium conditions. Cell migration ability was monitored by detecting the migration distance.

### Western blot

Total protein was isolated by using RIPA buffer (Beyotime), and then protein extracts were segregated by using 10% SDS-PAGE. Afterward, the samples were transferred to a PVDF membrane. Western blot was conducted in accordance with previous work [21]. The primary antibodies against cleaved caspase-3 (ab2302, 1:500), MMP9 (ab228402, 1:1000), GAPDH (ab9485, 1:2500), YY1 (ab109228, 1:1000) and secondary antibodies (ab205718, 1:2000) were all procured from Abcam (Cambridge, MA, USA). Finally, the enhanced chemiluminescence method (Beyotime) was used to measure the protein signals.

### Enzyme-linked immunosorbent assay (ELISA)

The cell supernatant of RA-FLSs was acquired and the expression levels of interleukin-6 (IL-6) and tumor necrosis factor-α (TNF-α) were examined by ELISA kits (Invitrogen).

### Dual-luciferase reporter and RNA immunoprecipitation (RIP) assay

The target between miR-204-5p with circ_0083964 or YY1 was predicted by StarBase 2.0 software (starbase.sysu.edu.cn). Wild-type or mutant sequences of circ_0083964 (WT-circ_0083964, MUT-circ_0083964), or YY1 3'UTR (WT-YY1 3'UTR, MUT-YY1 3'UTR) were cloned into the pmirGLO vector (Promega, Madison, WI, USA). Later on, cells were co-transfected with the reporter vectors and miR-204-5p mimic or control. Luciferase activities were measured via the dual-luciferase assay system (Promega).

Lysate of RA-FLSs was co-treated with magnetic beads coated with Ago2 or IgG antibodies (Abcam). The RNA was harvested and performed for qRT-PCR analysis.

### Statistical analysis

GraphPad Prism 7.0 software (GraphPad, La Jolla, CA, USA) was utilized to evaluate the data and the results were exhibited as mean ± standard deviation. The correlations were assessed via Pearson correlation analysis. *P* < 0.05 was considered a significant difference. Comparisons were estimated using Student’s *t*-test or one-way ANOVA.

## Results

### Circ_0083964 level was elevated in RA synovial tissues and RA-FLSs

**Fig. 1:**
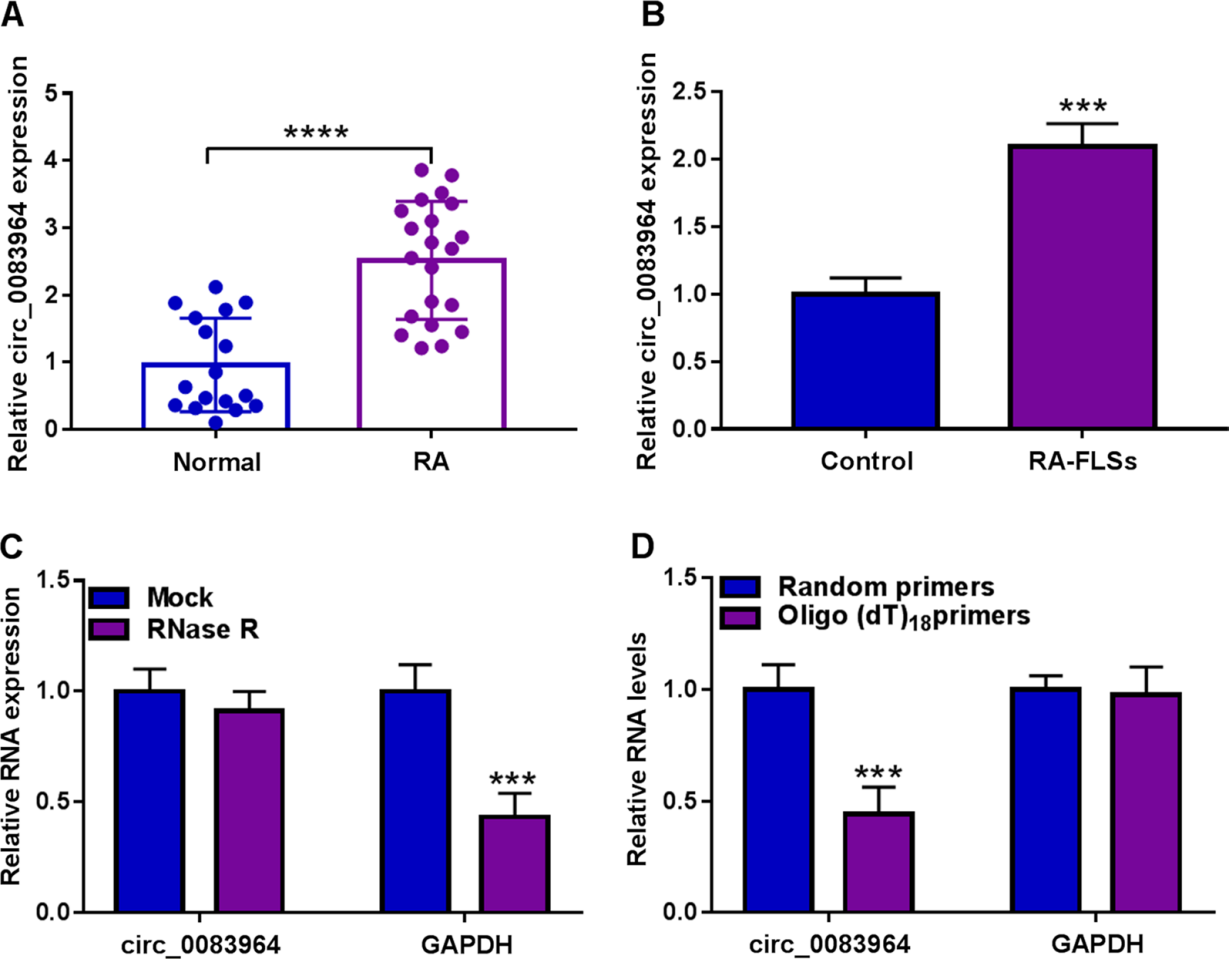
Circ_0083964 was upregulated in RA synovial tissues and RA-FLSs. **A** Relative circ_0083964 level was counted by qRT-PCR in RA synovial tissues (*n* = 17) and normal tissues (*n* = 21). **B** The expression of circ_0083964 was calculated by qRT-PCR in RA-FLSs and FLSs cells. **C** The stability of circ_0083964 was estimated by RNase R. **D** The level of circ_0083964 and GAPDH in reverse transcription was estimated by utilizing Random and Oligo(dT)18 primers. ***P < 0.001, ****P < 0.0001

The level of circ_0083964 was evaluated in RA synovial tissues and RA-FLSs by qRT-PCR. The results identified that circ_0083964 was upregulated in RA synovial tissues and RA-FLSs compared with normal synovial tissues and FLSs (Fig. 1A, B). Meanwhile, the circular characteristic of circ_0083964 was monitored by RNase R exposure, and the results found that circ_0083964 could resist RNase R digestion than linear RNA GAPDH (Fig. 1C). Furthermore, reverse transcription experiments were conducted by using random and oligo (dT) 18 primers, and the results proved the abundance of circ_0083964 was lower than linear transcript (Fig. 1D). Meanwhile, qRT-PCR was applied with genomic DNA (gDNA) and cDNA as templates, and GAPDH as a negative control. Agarose gel electrophoresis verified that circ_0083964 was only amplified by divergent primers in cDNA while GAPDH was amplified by convergent primers in both cDNA and gDNA (Additional file 1: Fig. S1A). The reverse splicing site of circ_0083964 was further demonstrated by utilizing Sanger sequencing (Additional file 1: Fig. S1B), confirming that circ_0083964 possessed a circular structure. Taken together, these data suggested circ_0083964 was increased in RA synovial tissues and RA-FLSs.

### Circ_0083964 silencing retarded RA progression

**Fig. 2:**
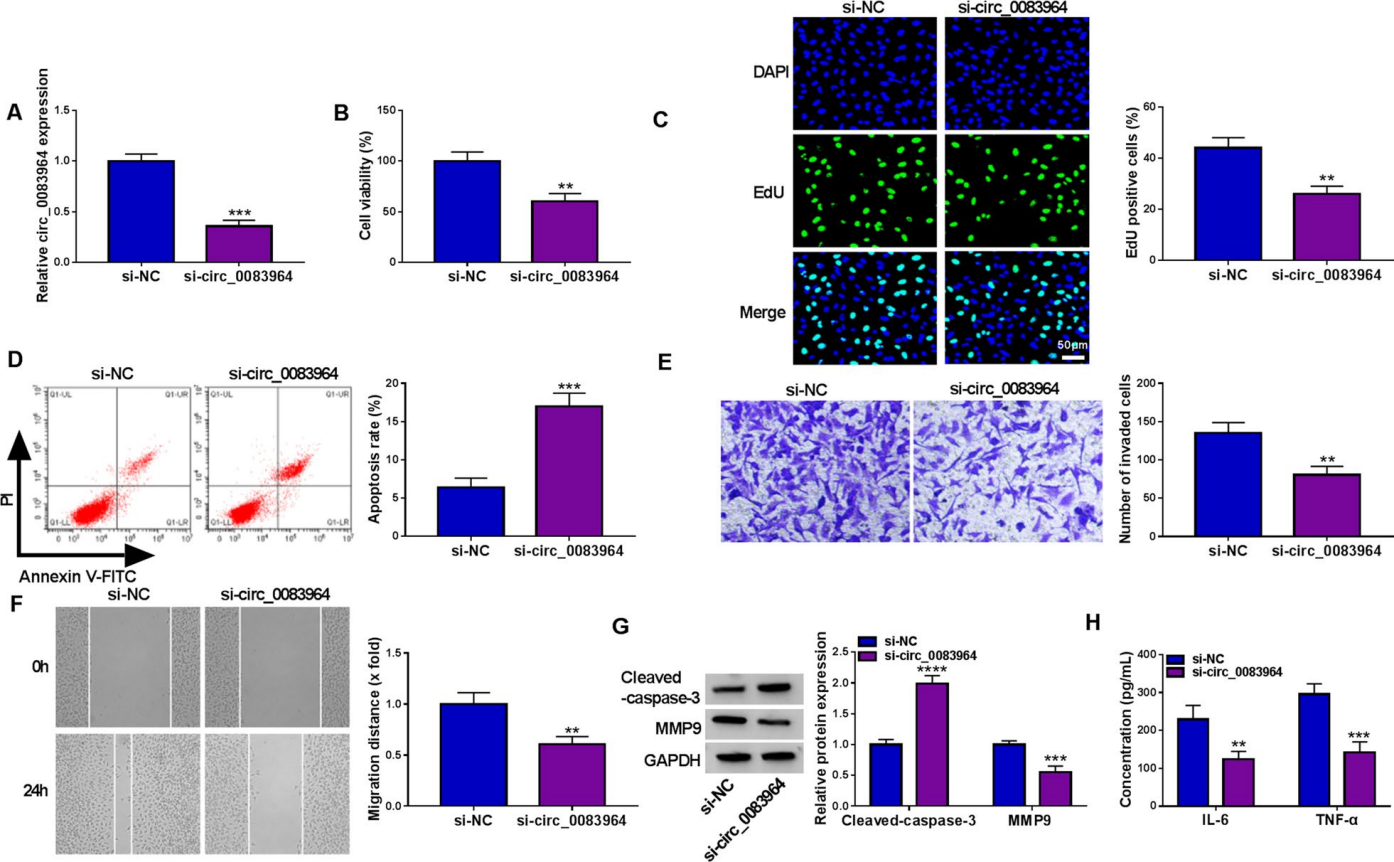
Circ_0083964 downregulation impeded cell proliferation, invasion, migration and inflammatory response in RA-FLSs. **A** The level of circ_0083964 was examined by qRT-PCR in RA-FLSs transfected with si-NC or si-circ_0083964. **B**–**H** RA-FLSs were divided into 2 groups: si-NC and si-circ_0083964. (B-F) Cell viability (**B**), EdU-positive cells (**C**), apoptosis (**D**), invasion (**E**) and migration (**F**) in RA-FLSs were evaluated by MTT assay, EdU assay, flow cytometry, transwell assay and wound-healing assay, individually. **G** The protein expression of cleaved caspase-3 and MMP9 was investigated by western blot in RA-FLSs. **H** ELISA assay was employed to examine the concentrations of IL-6 and TNF-α in RA-FLSs. **P < 0.01, ***P < 0.001, ****P < 0.0001

The qRT-qPCR assay demonstrated that the level of circ_0083964 was drastically reduced in RA-FLSs by transfecting si-circ_0083964 compared with si-NC group (Fig. 2A). In functional experiments, we confirmed that interference of circ_0083964 reduced the viability and proliferation of RA-FLSs (Fig. 2B, C). The apoptotic rate of RA-FLSs was strikingly elevated in si-circ_0083964 compared with si-NC group (Fig. 2D). The invasion and migration abilities of RA-FLSs were apparently restrained after circ_0083964 silencing (Fig. 2E, F). Furthermore, apoptosis and metastasis-related markers (cleaved caspase-3 and MMP9) were assessed via western blot assay, and the results manifested that circ_0083964 deficiency increased the cleaved caspase-3 level and reduced the level of MMP9 (Fig. 2G). In addition, the concentrations of IL-6 and TNF-α in RA-FLSs were evidently decreased after circ_0083964 knockdown (Fig. 2H). Overall, these results reflected that circ_0083964 downregulation constrained RA development.

### Circ_0083964 directly interacted with miR-204-5p

**Fig. 3:**
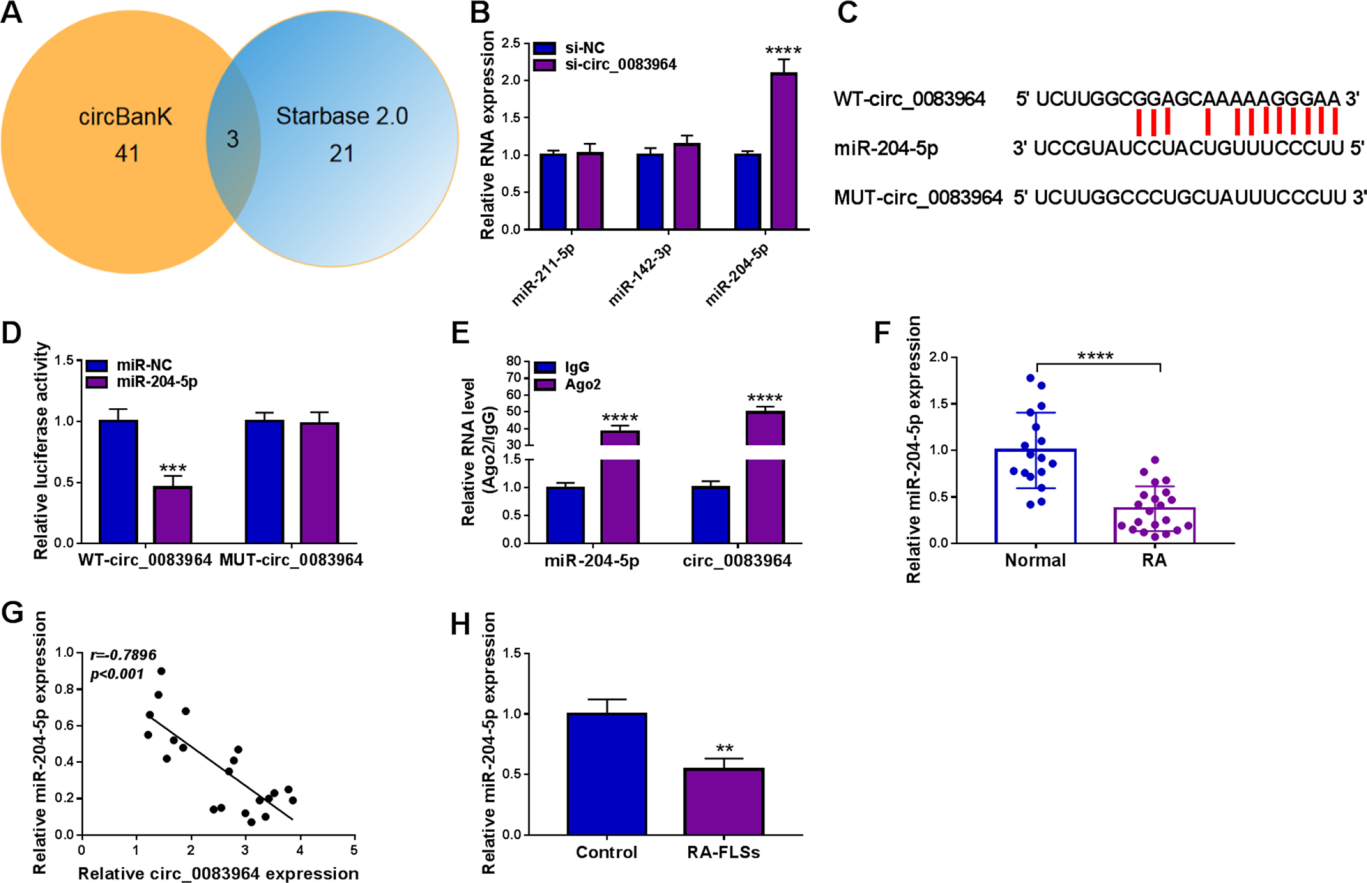
MiR-204-5p was a direct target of circ_0083964 in RA-FLSs. **A** StarBase and circBank databases showed three miRNAs that were targeted by circ_0083964. **B** The effect of circ_0083964 knockdown on the expression of three miRNAs was examined by qRT-PCR. **C** The binding regions with miR-204-5p in circ_0083964 were shown. **D**, **E** Dual-luciferase reporter assay and RIP assay were applied to analyze the target interaction between circ_0083964 and miR-204-5p. **F** The expression of miR-204-5p was examined in RA synovial tissues and normal tissues by qRT-PCR. **G** The correlation between the level of circ_0083964 and miR-204-5p was explored by Pearson correlation analysis. **H** The miR-204-5p level was estimated by qRT-PCR in RA-FLSs and control cells. **P < 0.01, ***P < 0.001, ****P < 0.0001

Venn diagram showed that StarBase 2.0 and circBanK databases had three same targeted miRNAs, and miR-204-5p was responsive to circ_0083964 deficiency (Fig. 3A, B), and circ_0083964 sequences were exhibited to have the miR-204-5p binding sites (Fig. 3C). Subsequently, dual-luciferase reporter assay and RIP assay were executed to demonstrate the associative relation, and the results showed miR-204-5p conspicuously reduced the luciferase activity in WT-circ_0083964 group, while luciferase activity was not changed in MUT-circ_0083964 group (Fig. 3D). In addition, circ_0083964 and miR-204-5p were both enriched in Ago2 group (Fig. 3E). The expression of miR-204-5p was obviously reduced in RA synovial tissues relative to normal tissues (Fig. 3F), and miR-204-5p level in RA synovial tissues was negatively correlated with circ_0083964 level (Fig. 3G). Compared with control cells, miR-204-5p expression was specially downregulated in RA-FLSs (Fig. 3H). These results suggested that circ_0083964 negatively regulated miR-204-5p level in RA-FLSs through binding to it.

### MiR-204-5p inhibition reversed the impact of circ_0083964 knockdown in RA progression

**Fig. 4:**
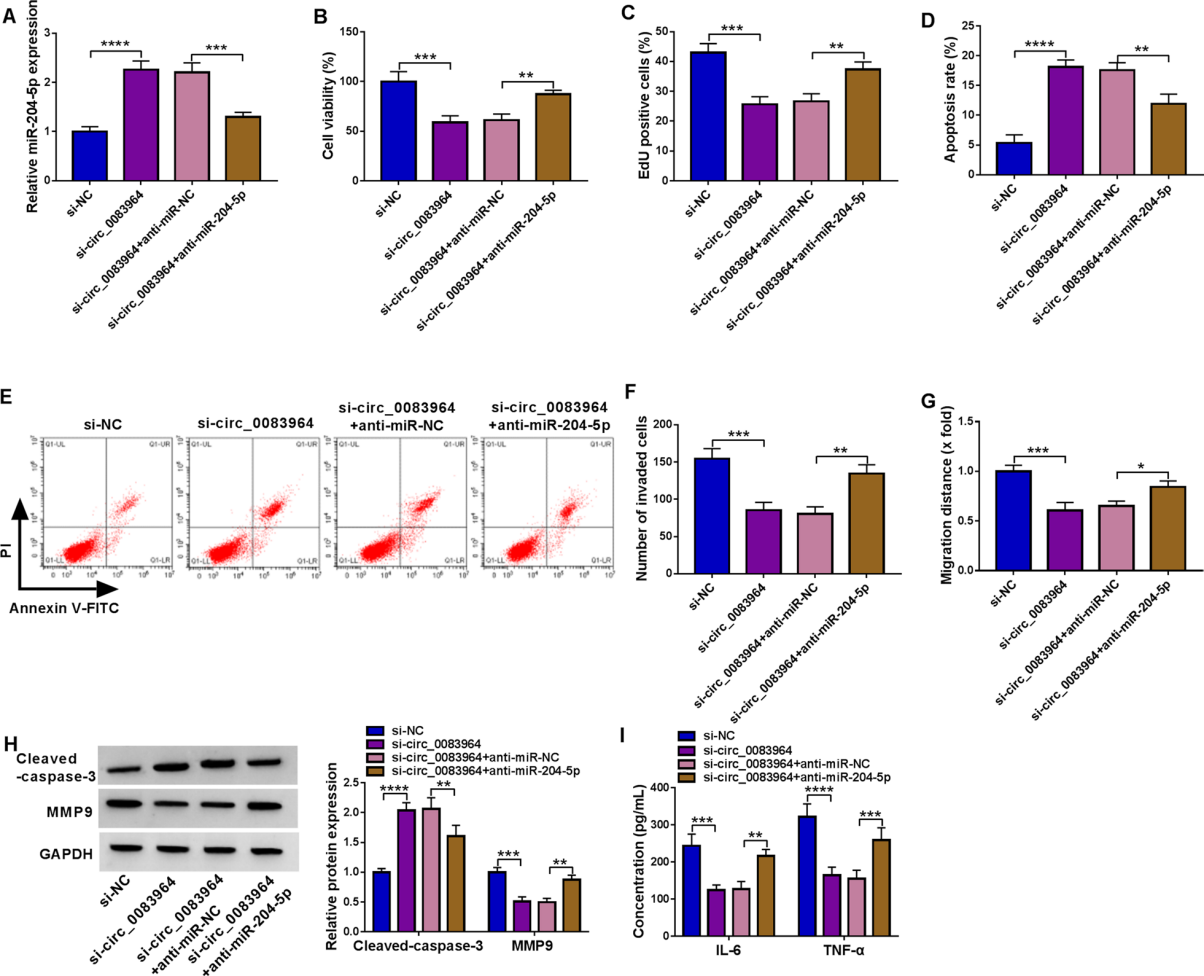
MiR-204-5p inhibition overturned the effects caused by circ_0083964 knockdown in RA-FLSs. **A–I** RA-FLSs were transfected with si-NC, si-circ_0083964, si-circ_0083964 + anti-miR-NC or si-circ_0083964 + anti-miR-204-5p. **A** The expression of miR-204-5p was detected by qRT-PCR. **B–G** CCK-8 assay, EdU assay, flow cytometry, transwell assay and wound-healing assay were used to assess cell viability, proliferation, apoptosis, invasion and migration, respectively. **H** The protein levels of cleaved caspase-3 and MMP9 were detected by western blot. **I** The levels of IL-6 and TNF-α in RA-FLSs were evaluated by ELISA assay. *P < 0.05, **P < 0.01, ***P < 0.001, ****P < 0.0001

Then, we explored whether circ_0083964 regulated RA development by absorbing miR-204-5p. Interference of circ_0083964 increased miR-204-5p level in RA-FLSs, and miR-204-5p expression was overturned by the anti-miR-204-5p introduction (Fig. 4A). Function experiments displayed that miR-204-5p deficiency could abrogate the impacts of circ_0083964 inhibition on cell viability (Fig. 4B), EdU-positive cells (Fig. 4C), apoptosis (Fig. 4D, E), invasion (Fig. 4F) and migration (Fig. 4G) in RA-FLSs. In addition, the effects of circ_0083964 knockdown on cleaved caspase-3 and MMP9 protein levels, as well as the release of inflammatory factor were counteracted by the addition of anti-miR-204-5p (Fig. 4H, I). All data identified that circ_0083964 regulated RA progression by regulating the expression of miR-204-5p.

### MiR-204-5p directly targeted YY1

**Fig. 5:**
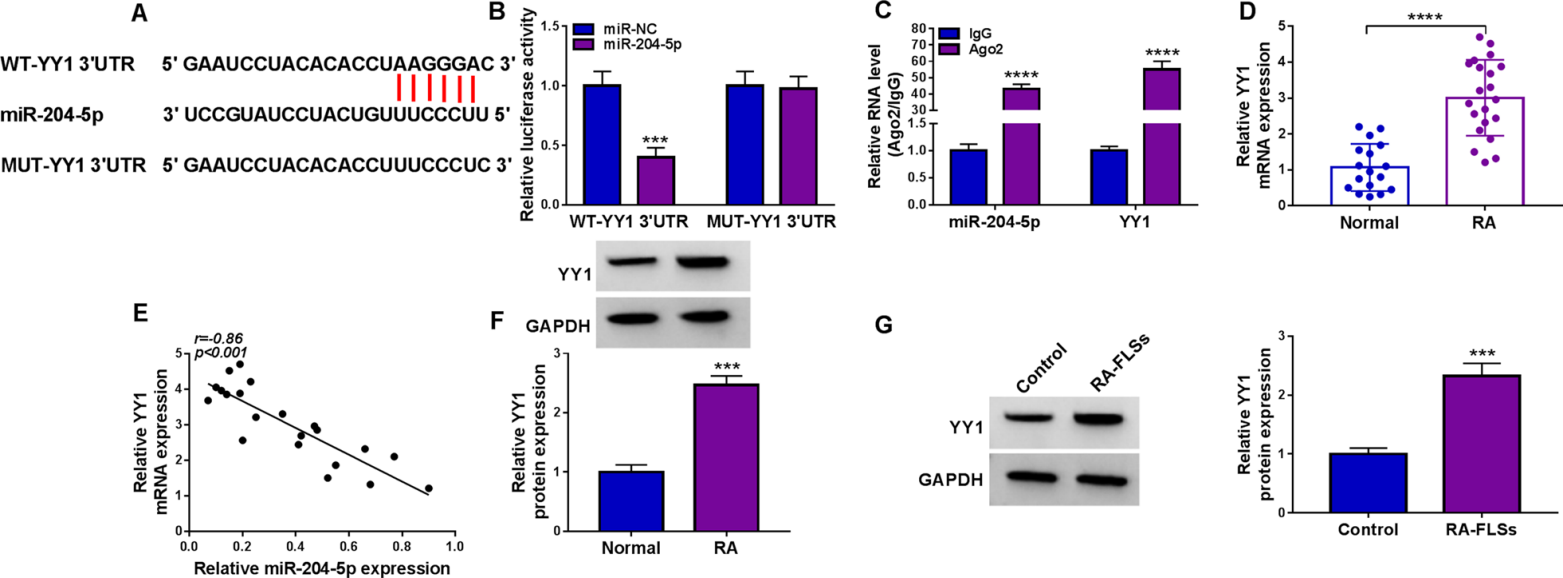
YY1 was a direct target of miR-204-5p. **A** StarBase dataset was used to predict the binding region between YY1 and miR-204-5p. **B**, **C** The target interaction between miR-204-5p and YY1 was tested by dual-luciferase reporter assay and RIP assay. **D** The mRNA level of YY1 was obtained in RA synovial tissues by qRT-PCR. **E** The linear relationship between the expression of YY1 and miR-204-5p was examined by Pearson correlation analysis. **F**, **G** Western blot assay was applied to illustrate the protein expression of YY1 in RA synovial tissues and RA-FLSs. ***P < 0.001, ****P < 0.0001

**Fig. 6:**
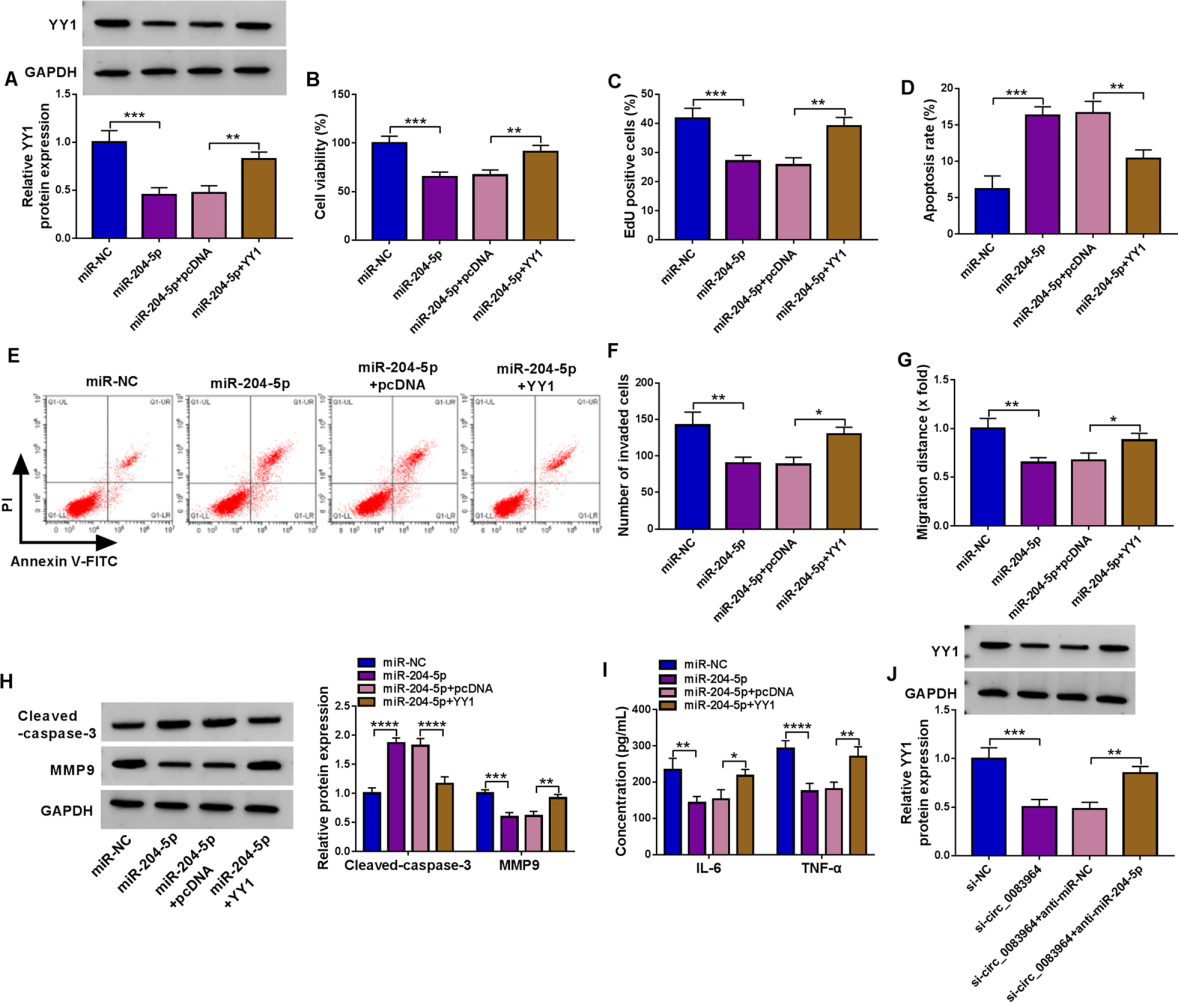
MiR-204-5p constrained RA progression via downregulating YY1 in RA-FLSs. **A**–**I** Transfection of miR-NC, miR-204-5p, miR-204-5p + pcDNA, or miR-204-5p + YY1 was performed in RA-FLSs. (A) YY1 level was gauged by western blot. **B**–**G** CCK-8 assay, EdU assay, flow cytometry, transwell assay and wound-healing assay were implemented to reveal cell viability, proliferation, apoptosis, invasion and migration, individually. **H** The protein quantification of cleaved caspase-3 and MMP9 was performed by western blot. **I** Inflammatory response was measured by ELISA assay. **J** The impact of the transfection of si-NC, si-circ_0083964, si-circ_0083964 + anti-miR-NC or si-circ_0083964 + anti-miR-204-5p on the expression of YY1 in RA-FLSs was analyzed by western blot. *P < 0.05, **P < 0.01, ***P < 0.001, ****P < 0.0001

To reveal the target gene of miR-204-5p, the StarBase software was used. StarBase predicted that miR-204-5p had many target mRNAs. Through literature research, we searched for mRNAs that were highly expressed in RA and promoted the progression of RA. QRT-PCR experimental analysis found that miR-204-5p could significantly affect the expression of YY1. Consequently, the function of miR-204-5p/YY1 in RA was further investigated, and the data were displayed in Additional file 2: Fig. S2. The targeting sites between miR-204-5p and YY1 were shown in Fig. 5A. Furthermore, miR-204-5p mimic drastically reduced the luciferase activity of WT-YY1 3’UTR group but did not affect the MUT-YY1 3’UTR group (Fig. 5B). Besides that, the enrichments of YY1 and miR-204-5p were drastically augmented in Ago2 by RIP assay (Fig. 5C). Furthermore, we demonstrated that mRNA expression of YY1 was elevated in RA synovial tissues, and its expression was negatively correlated with miR-204-5p expression (Fig. 5D). Similarly, the protein expression of YY1 in RA synovial tissues and RA-FLSs was also substantially increased compared to the corresponding controls (Fig. 6F, G). All in all, the above results suggested that YY1 could be targeted by miR-204-5p.

### Upregulation of YY1 mitigated the effects of miR-204-5p restoration on proliferation, metastasis and inflammatory response in RA-FLSs

To further estimate the molecular mechanism of miR-204-5p in RA-FLSs, cells were transfected with miR-NC, miR-204-5p, miR-204-5p + pcDNA, or miR-204-5p + YY1. Western blot assay manifested that overexpression of miR-204-5p resulted in notable reduced expression of YY1 protein, while upregulation of YY1 abated this effect (Fig. 6A). The functional experiments revealed that miR-204-5p overexpression hampered cell viability, and facilitate apoptosis in RA-FLSs, which were receded by the restored YY1 expression (Fig. 6B–E). Furthermore, miR-204-5p overexpression distinctly impeded the invasion and migration of RA-FLSs, while these influences were weakened by elevating YY1 (Fig. 6F, G). Moreover, the effects of miR-204-5p on the protein expression of cleaved caspase-3 and MMP9 and inflammatory response in RA-FLSs also were ameliorated by YY1 overexpression (Fig. 6H, I). These results disclosed that miR-204-5p introduction restrained cell proliferation, metastasis and inflammatory response of RA-FLSs by downregulating YY1. More importantly, circ_0083964 deficiency led to a reduction in YY1 level both at protein levels, which was counteracted by the addition of anti-miR-204-5p in RA-FLSs (Fig. 6J), indicating the feedback loop of circ_0083964/miR-204-5p/YY1 in RA.

## Discussion

The pathogenesis of RA is complex [22]. A large amount studies have verified that the progression of RA is associated with immune disorders and persistent inflammation. In addition, the tumorlike biological characteristics of RA-FLSs were found to be closely related to the pathogenesis of RA [23]. At present, the diagnosis and treatment of RA are still facing great challenges. Therefore, an in-depth understanding of the pathogenesis of RA is dramatically pivotal for exploring new therapeutic targets of RA.

CircRNAs have been found to serve as a vital part in the development of RA [24]. However, the mechanisms of most circRNAs in the progress of RA have not been fully elucidated. Our work proved the potential role of circ_0083964 in RA and found that circ_0083964 was elevated in RA synovial tissues and RA-FLSs, which was in accordance with previous studies. The results of the functional experiment verified that the circ_0083964 deficiency could remarkably curb the proliferation, invasion, migration and inflammatory response of RA-FLSs and pronouncedly trigger the apoptosis of RA-FLSs, confirming that circ_0083964 may expedite RA progression.

Subsequently, circ_0083964 was identified to serve as a sponge of miR-204-5p in RA-FLSs, and the interference of circ_0083964 confined RA development, while this effect was neutralized by miR-204-5p downregulation. MiRNAs are short RNA composed of 18–25 nucleotides that regulate various cellular functions by regulating the levels of target genes [25]. Many studies have disclosed that miR-204-5p has anti-proliferation and anti-metastasis effects in the development of a variety of tumors. MiR-204-5p hampered the proliferation and invasion of laryngeal squamous cell carcinoma by reducing the abundance of SEMA4B [26]. MiR-204-5p restrained the malignant progression of retinoblastoma by regulating ROCK1 expression [27]. And miR-204-5p also blocked gastric cancer cell proliferation and metastasis [28]. These results proved that miR-204-5p has the property of inhibiting the tumorlike behavior of cells. Furthermore, Xiao et al. [19] proposed that miR-204-5p was reduced in RA synovial tissues, and miR-204-5p overexpression effectively impeded cell proliferation and inflammatory response and triggered apoptosis of RA-FLSs. Similarly, our work also demonstrated the level of miR-204-5p was decreased in RA synovial tissue and RA-FLSs and revealed the targeting relationship between miR-204-5p and circ_0083964. Besides that, the rescue experiments validated that circ_0083964 mediated the proliferation, apoptosis, invasion, migration and inflammatory response of RA-FLSs through absorbing miR-204-5p. Thus, we concluded that circ_0083964 influenced RA progression via sponging miR-204-5p in RA-FLSs.

The binding relationship between YY1 and miR-204-5p was verified in RA-FLSs. YY1 is a ubiquitously expressed transcription factor that interacts with a variety of cofactors to regulate cellular biological processes. Moreover, YY1 could activate the inflammatory process in the immune system [29]. At present, a series of studies have uncovered that YY1 participated in the development of RA. Cai et al. [30] demonstrated that miR-449a suppressed RA-FLSs proliferation, migration and inflammatory processes by reducing the expression of YY1. Wand et al. [31] suggested that NEAT1 reinforced the proliferation and constrained the apoptosis in RA-FLSs by regulating miR-410-3p/YY1 axis. Herein, YY1 was manifested to be upregulated in RA synovial tissues and RA-FLSs. Additionally, miR-204-5p negatively regulated the YY1 level. MiR-204-5p overexpression hampered the RA progression, which was effectively rescued by forcing the expression of YY1, suggesting that miR-204-5p hindered RA progression partly by reducing the abundance of YY1. More importantly, circ_0083964 inhibition decreased YY1 expression, and the transfection of anti-miR-204-5p partially overturned the level of YY1, reflecting that circ_0083964 partially upregulated YY1 expression by targeting miR-204-5p. In terms of the limitations of this study, whether circ_0083964 depletion could play a role in inhibiting RA progression in vivo studies needs to be further explored. We will also pay further attention to the in vivo research progress of circ_0083964.

**Fig. 7:**
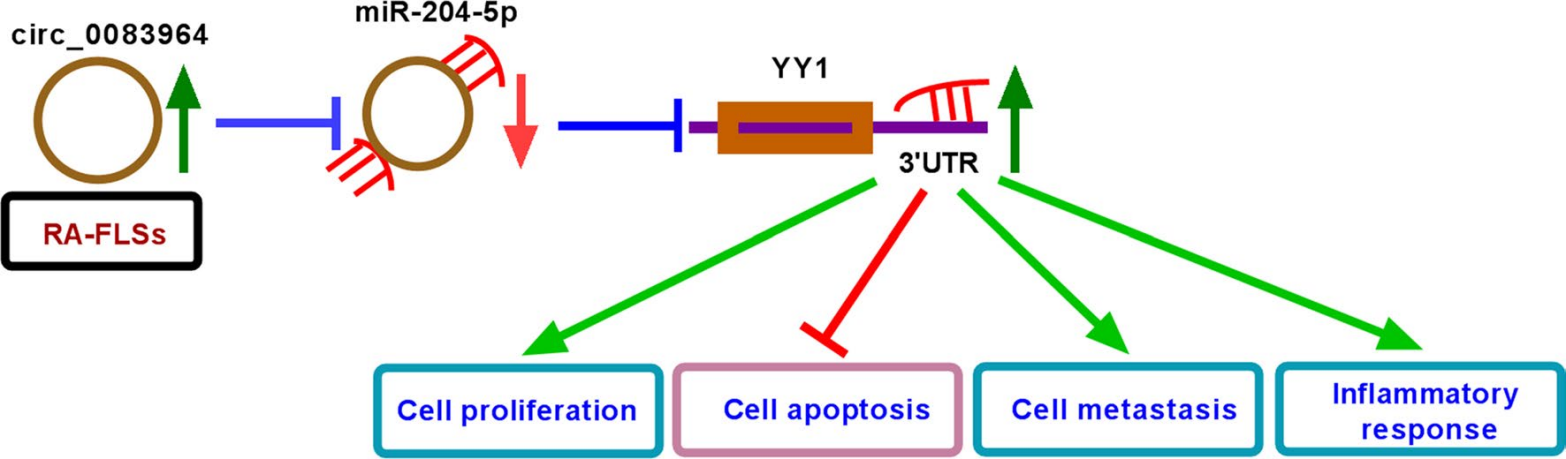
Summary diagram of this study

Collectively, circ_0083964 knockdown reduced YY1 level via adsorbing miR-204-5p, thereby restraining RA progression (Fig. 7). The work provided a novel mechanism by which circ_0083964 regulated RA development.

## Supplementary Information

**Additional file 1: Fig. S1.** **A** Agarose gel electrophoresis identified the existence of circ_0083964 in RA-FLSs. **B** Detection of the cyclization site by Sanger sequencing.

**Additional file 2: Fig. S2.** The mRNA expression was detected by qRT-PCR. **P < 0.01, ***P < 0.001, ****P < 0.0001.

## Data Availability

Not applicable.
