# Supplementary figures and images for: Circ_0083964 knockdown impedes rheumatoid arthritis progression via the miR-204-5p-dependent regulation of YY1
Source: J Orthop Surg Res. 2022 Dec 22;17:558. doi: 10.1186/s13018-022-03353-5 (PMC9773446; doi:10.1186/s13018-022-03353-5)

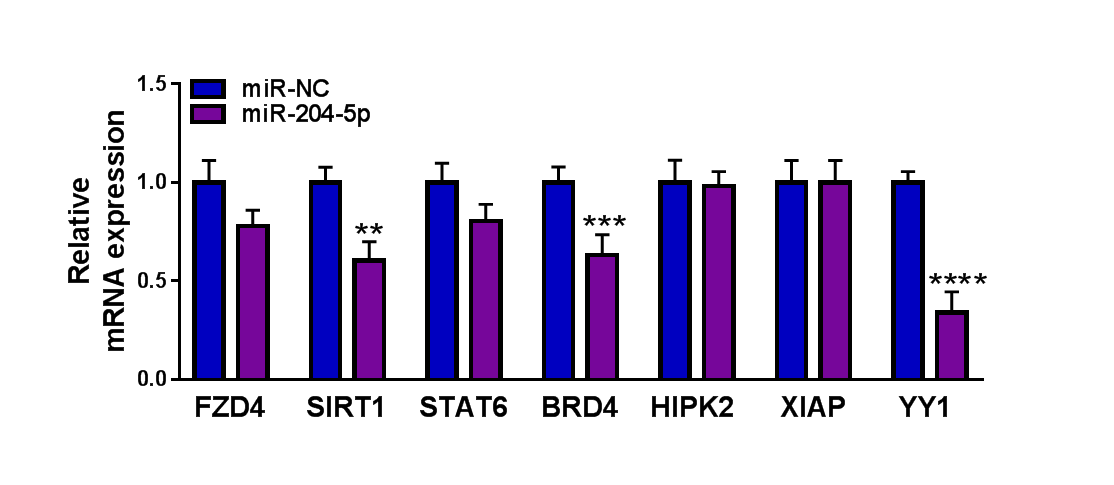

Supplement: Supplementary file 1 — Additional file 1: Fig. S1. A Agarose gel electrophoresis identified the existence of circ_0083964 in RA-FLSs. B Detection of the cyclization site by Sanger sequencing. [file 13018_2022_3353_MOESM1_ESM.tif]

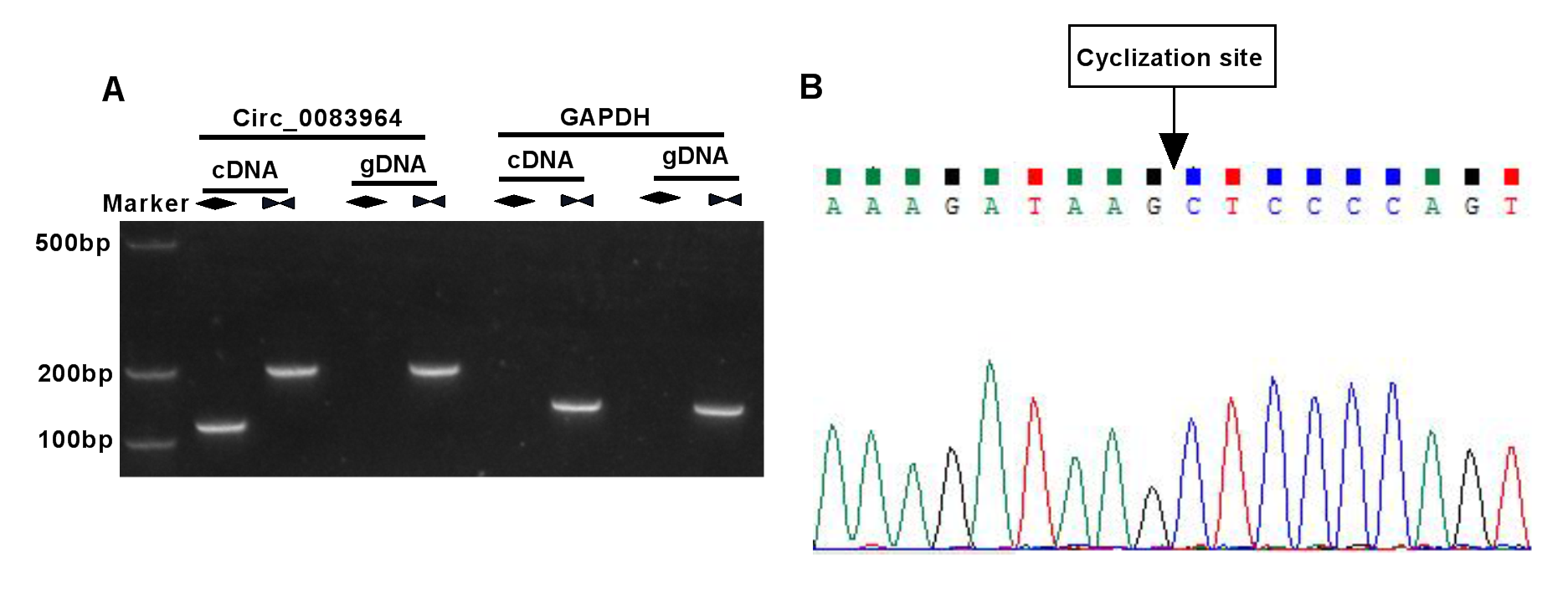

Supplement: Supplementary file 2 — Additional file 2: Fig. S2. The mRNA expression was detected by qRT-PCR. **P < 0.01, ***P < 0.001, ****P < 0.0001. [file 13018_2022_3353_MOESM2_ESM.tif]
